# Knowledge, attitudes, and skills towards evidence-based medicine among Palestinian undergraduate medical students: a cross-sectional study

**DOI:** 10.1186/s12909-026-08750-6

**Published:** 2026-02-05

**Authors:** Hamza Karmi, Hebatallah Qawasmeh, Rasha K. Ahmad, Suhaib Alkhateeb, Firas Awawdeh, Bashar Douden, Azzam Zrineh, Rami Akwan

**Affiliations:** 1 Faculty of Medicine, Al-Quds University, Jerusalem, Palestine; 2 Faculty of Medicine, Palestine Polytechnic University, Hebron, Palestine; 3 Faculty of Medicine and Health Sciences, An-Najah National University, Nablus, Palestine; 4 Faculty of Graduate Studies, Arab American University, Jenin, Palestine; 5 Syrian Private University, Damascus, Syria

**Keywords:** Attitude, Evidence-based medicine, Medical education, Palestine, Skills, Source of information, Undergraduate students.

## Abstract

**Background:**

Evidence-based medicine (EBM) has been increasingly emphasized within undergraduate medical education; however, its implementation remains inconsistent across many low- and middle-income countries. In Palestine, restricted access to scientific resources, inconsistent mentorship, and limited institutional funding and support may have affected students’ competencies in EBM. This study evaluated the knowledge, attitudes, skills, and information-seeking behaviours related to EBM among undergraduate medical students in the West Bank.

**Methods:**

A cross-sectional study was conducted between 14 September 2025 and 1 November 2025, among second to sixth-year medical undergraduates at five Palestinian universities. The questionnaire was distributed through convenience sampling and included five sections: demographics, information-seeking behaviours, EBM knowledge, skills, and students’ attitudes towards it. Analyses were performed using Jamovi 2.6.44 statistical package.

**Results:**

A total of 709 students participated, with a mean age of 21.3 years, of whom 37% had trained in EBM. Practical workshops were the most preferred learning modality (31%), while medical books were the most frequently used source of information (83.6%). Regarding search engines, PubMed, Google and Google scholar were the most accessed. Attendance at biostatistics or research methodology courses was significantly associated with prior EBM training. Overall, students showed positive attitudes towards EBM, and average self-rated skills. However mean score for each skill was significantly higher in EBM-trained undergraduates compared to non-trained peers (*p *value < 0.001). Understanding of EBM-related terms varied, with participants being more familiar with basic terms than advanced terms, such as heterogeneity, and number needed to treat (NNT), and prior EBM training was consistently associated with higher knowledge scores for key terms. As for research barriers, the majority reported limited access to paid databases, limited funding, and inconsistent mentorship.

**Conclusion:**

Palestinian medical students held positive stance towards evidence-based medicine, recognizing its clinical value. However, their skills and terminology knowledge were still somehow limited. Integrating EBM training into curriculum, enhancing faculty support, and expanding access to resources can strengthen students’ competency and prepare them for evidence-based clinical practice.

## Introduction

Evidence-based medicine (EBM) refers to the application of the best available scientific evidence, clinical expertise, and patient values to inform clinical decision-making [1]. In recent years, EBM has evolved from a conceptual framework into an integral component of healthcare education and routine practices. It supports the formulation of focused clinical questions, the critical appraisal of relevant research, and the integration of evidence-based information into patient-centered care [1].

Furthermore, International organizations and guideline-developing committees have strengthened evidence-based decision-making by employing standardized frameworks such as the Grading of Recommendations, Assessment, Development and Evaluation (GRADE). This approach ensures that healthcare systems are provided with consistent, clear, and high-quality clinical recommendations supported by methodological rigor [2].

To guide the integration of EBM into healthcare curricula, a Delphi survey informed by a large systematic review developed a consensus statement covering 68 competencies to support EBM teaching. These competencies were based on the five domains of evidence-based practice (EBP), which are Ask, Acquire, Appraise, Interpret, Apply, and Evaluate [3].

In EBM education research, learner competence is commonly defined and assessed across four domains: knowledge, skills, attitudes, and contextual resources or barriers. Knowledge refers to understanding EBM concepts and terminology and is often assessed through self-reported familiarity and the ability to explain key terms [4]. Skills reflect learners’ ability to perform core EBM steps, including formulating answerable clinical questions, searching for evidence, and critically appraising research. Attitudes capture the perceived value of EBM and the intention to apply it in clinical practice [5]. Resources and barriers represent contextual determinants that enable or impede EBM learning and use, including time constraints, access to evidence sources, and the availability of training, mentorship, and institutional support [6, 7].

Experimental studies have been conducted to assess the benefits of integrating EBM teaching into medical curricula and reported improvements in knowledge and skills of clinical-year medical students [8, 9]. A meta-regression analysis among undergraduate nursing students further supported these findings [10].

Nonetheless, despite EBM advantages and global adoption, low- and middle-income countries (LMICs) continue to face several challenges in adopting it into educational and clinical settings. These include limited resources, inadequate institutional support, and other structural and process-related barriers, which prevent students and clinicians from effectively implementing evidence-based practices [11]. Studies conducted in Sudan and Ethiopia have highlighted gaps in EBM knowledge and skills among undergraduate students, attributed to such barriers [12, 13].

In Palestine, EBM research has largely focused on practicing clinicians. A Gaza survey assessed physicians’ awareness, knowledge, and attitudes toward EBM [14], while West Bank studies among nurses evaluated EBM knowledge, attitudes, practice, and barriers to implementation [15]. EBM competency domains have also been measured among Palestinian nursing students [16]. However, undergraduate medical education studies have addressed research training perceptions rather than providing a competency-based, multi-domain assessment of EBM knowledge, task-based competencies, attitudes, and enabling resources among Palestinian medical students [17].

In this context, the present study evaluated EBM-related knowledge, self-reported skills, attitudes, and perceived barriers among undergraduate medical students across Palestinian medical schools. Baseline patterns were described, and differences by prior EBM training were examined using item-level analyses. Barriers and resource constraints were also identified to inform curriculum integration and targeted educational support.

## Method 

### Study design and population

This was a cross-sectional study designed to assess the knowledge, skills, attitudes, and information-seeking behaviours related to EBM among undergraduate medical students across the West Bank.

Our sample consisted of undergraduate medical students (aged 19 years and above) who were registered in MD programmes at major Palestinian universities during the data collection period. These institutions included An-Najah National University (Nablus), Arab American University (Jenin), Al-Quds University (Jerusalem), Palestine Polytechnic University (Hebron), and Hebron University (Hebron), representing northern, central, and southern academic settings in the West Bank.

### Sample size calculation

The sample size was calculated using the ‘OpenEpi’ online calculator [18], assuming a 95% confidence level, 50% expected proportion to account for maximum variability, and a 5% margin of error. The minimum required sample size was 384 students. To enhance statistical power and ensure representation across universities and academic years, a minimum of 500 respondents was sought.

### Inclusion and exclusion criteria

#### Inclusion criteria

The study included undergraduate Palestinian medical students aged 19 years or older, enrolled in the second through sixth academic years at any of the participating universities. Only those who were able to understand and complete the online questionnaire and provide informed consent were included.

#### Exclusion criteria

First-year students were excluded, as they had not yet been introduced to the concept of evidence-based medicine. Furthermore, individuals who declined to provide informed consent were excluded.

### Data collection instruments

The questionnaire comprised two primary sections. The first section collected participant characteristics and academic/research background information (e.g., age, gender, academic level, prior courses in biostatistics and research methodology, and prior research exposure/engagement) and was included for descriptive purposes.

The second section was adopted from the previously published EBM questionnaire used by Hasabo et al. [19] and comprised four domains assessing: [1] information-seeking behaviours [2], evidence-based medicine (EBM) competencies on a 5-point Likert scale (1 = poor, 5 = advanced) [3], attitudes towards evidence-based practice on a scale of 1 to 5, with 1 being “strongly disagree” and 5 being “strongly agree”, and [4] knowledge of core EBM terminology using a 5-point Likert scale (5 = understand and can explain to others; 4 = some understanding; 3 = do not understand but would like to understand; 2 = do not understand and do not consider it useful to understand; 1 = no idea). The domain items and response options were used without modification.

Skills, attitudes, and terminology knowledge domains were coded according to their ordered response anchors and analysed at the item level; composite domain scores were not calculated by summing or averaging items. Item responses were summarised as mean ± standard deviation (SD), with higher scores consistently indicating greater self-reported competence, more favourable attitudes, and higher conceptual understanding, respectively.

### Validity and reliability

Content validity and clarity were reviewed by medical education experts to ensure cultural appropriateness and pilot testing of 30 students was conducted to confirm comprehensibility and feasibility. Internal consistency was assessed using Cronbach’s alpha, which showed good reliability across the subscales, ranging between 0.864 and 0.920. These were comparable to the original study, which had Cronbach’s alpha values ranging from 0.71 to 0.89 [5]. The questionnaire was administered in English, the language of instruction in Palestinian medical education.

### Data collection procedure

Given the absence of a centralized sampling frame and the routine use of digital communication channels among Palestinian medical students, a non-probability convenience sampling approach was used to recruit participants across the participating universities. Data were collected between 14 September 2025 and 1 November 2025 using an online questionnaire administered via Google Forms. The survey link was distributed to medical student groups at each university through WhatsApp, Facebook, Telegram, and Facebook Messenger. A standardized invitation message and an identical survey link were disseminated across all sites during the same recruitment period to ensure consistency.

To maximize participation, reminders were posted weekly through the same channels. Because dissemination occurred through open digital groups rather than controlled university email lists, the number of students exposed to the survey link could not be reliably enumerated; therefore, response rates stratified by university and academic year could not be calculated.

### Data management and statistical analysis

Jamovi statistical software (version 2.6.44) was employed to analyse the data. As all items were mandatory in Google Forms, no item-level missing data were observed. The final analytic sample comprised 709 respondents.

Continuous variables were reported as means and standard deviations (SD), while categorical variables were reported as frequencies and percentages. Normality of continuous data were assessed using Kolmogorov-Smirnov test, along with plot visualisation, which indicated a non-normal distribution for most numerical variables. Nonetheless, means (SD) were presented due to the narrow range of scales and for better comparison, as median and interquartile range were less informative in this context.

The Mann-Whitney U test was used to assess group differences between students with and without prior training in EBM for continuous variables; as for categorical variables, the Chi-square test was employed. The threshold for statistical significance was set at *p* value < 0.05.

### Ethical considerations

The study received ethical approval from the Institutional Review Board (IRB) at Al-Quds University (Ref No:586/REC/2025). Participation was voluntary and anonymous, and electronic informed consent was obtained from participants.

## Results 

A total of 709 undergraduate medical students from five Palestinian universities completed the survey; 262 (37.0%) reported prior EBM training. Overall, students reported generally positive attitudes toward EBM, but self-reported competencies were mostly average, with critical appraisal consistently emerging as the weakest skill area. Evidence acquisition behaviours relied heavily on PubMed/Medline and general search engines, while specialized evidence-based databases were rarely used, and several structural supports (notably mentorship, paid database access, and funding) were limited. Across domains, prior EBM training was consistently associated with more evidence-oriented information-seeking, higher self-reported skills, and higher conceptual understanding for most terms.

### Participants’ demographics

709 undergraduate medical students from five Palestinian universities with a mean age of 21.3 years participated in this cross-sectional study. 262 participants trained in EBM concepts and 447 participants having no prior training. Females comprised nearly two-thirds (65.9%, *n* = 467) of the sample and students from second to sixth years had relatively balanced representation.

Overall, academic level was significantly associated with EBM training (*p* value < 0.001). Nonetheless, participation in clinical training at hospitals was significantly associated with a lower proportion of students trained in evidence-based practice (*p *value < 0.001). Detailed baseline characteristics and comparisons are presented in Table 1.

**Table 1:** Baseline characteristics of medical students who completed the online survey in Palestinian universities (*N* = 709)

Variables	Descriptive	EBM Training		*p *value*
		Yes : 262(37%)	No : 447(63%)	
Age (years)	21.3 ± 1.57			
Gender				0.083
Male	242 (34.1%)	100(41.3%)	142(58.7%)	
Female	467 (65.9%)	162(34.7%)	305(65.3%)	
University Level				**< 0.001**
Second year	137 (19.3%)	29 (21.2%)	108 (78.8%)	
Third year	120 (16.9%)	45 (37.5%)	75 (62.5%)	
Fourth year	144 (20.3%)	64 (44.4%)	80 (55.6%)	
Fifth year	143 (20.2%)	51 (35.7%)	92 (64.3%)	
Sixth year	165 (23.3%)	73 (44.2%)	92 (55.8%)	
Received or attended any physical or online course in biostatistics (Yes)	327 (46.1%)	186 (56.9%)	141 (43.1%)	**< 0.001**
Received or attended any physical or online course in Research Methodology (Yes)	420 (59.2%)	218 (51.9%)	202 (48.1%)	**< 0.001**
Close family members working in healthcare (Yes)	335 (47.2%)	129 (38.5%)	206 (61.5%)	0.417
Internet access at University or home (Yes)	648 (91.4%)	238 (36.7%)	410 (63.3%)	0.686
Participation in any clinical training in a hospital during medical school years (Yes)	442 (62.3%)	199 (45%)	243 (55%)	**< 0.001**

Continuous data were presented as Mean ± SD, and categorical data were presented as frequencies and percentages

Percentages are row percentages (sum to 100% across training categories)

*Statistical tests performed: Chi-Square Test. Bold values indicate statistical significance (*p* < 0.05)

### Research engagement, resource access, and preferred learning methods

As shown in Table 2, research involvement was significantly associated with EBM training status (*p* < 0.001). Nearly half of the participants reported no prior research experience, while 29.9% reported involvement as authors, and 20.6% as data collectors. In addition, time spent reading medical literature was also significant between EBM groups (*p* = 0.01). Among students reading less than 1 h, 32.1% were trained in EBM while among 4–7 h and more than 7 h of reading per week, 36.2%, and 51.4%, respectively were EBM-trained.

**Table 2:** Research involvement, learning practices, and academic resource access among Palestinian medical students (*N* = 709)

Variable	*N* (%)	EBM training		*p* value*
		Yes : 262(37%)	No : 447(63%)	
Research involvement				**< 0.001**
Never	351 (49.5%)	104 (29.6%)	247 (70.4%)	
Author	212 (29.9%)	108 (50.9%)	104 (49.1%)	
Data collector	146 (20.6%)	50 (34.2%)	96 (65.8%)	
Time spent reading medical literature (per week)				**0.01**
Less than 1 h	396 (55.9%)	127(32.1%)	269 (67.9%)	
1–3 h	220 (31%)	96 (43.6%)	124 (56.4%)	
4–7 h	58 (8.2%)	21 (36.2%)	37 (63.8%)	
More than 7 h	35 (4.9%)	18 (51.4%)	17 (48.6%)	
Access to academic mentorship				**0.019**
Always	72 (10.2%)	30 (41.7%)	42 (58.3%)	
Sometimes	301 (42.5%)	128 (42.5%)	173 (57.5%)	
Rarely	195 (27.5%)	61 (31.3%)	134 (68.7%)	
No access	141 (19.9%)	43 (30.5%)	98 (69.5%)	
Preferred EBM learning method				0.122
Practical workshops	220 (31%)	81 (36.8%)	139 (63.2%)	
Online short courses	140 (19.7%)	57 (40.7%)	83 (59.3%)	
Lectures	136 (19.2%)	41 (30.1%)	95 (69.9%)	
Self-study	114 (16.1%)	38 (33.3%)	76 (66.7%)	
Guided research project	99 (14%)	45 (45.5%)	54 (54.5%)	
Career intention (definite academic/research career)	370 (52.2%)	145(39.2%)	225 (60.8%)	0.204
Access to paid academic databases (Yes)	129 (18.2%)	60 (46.5%)	69 (53.5%)	**0.013**
Availability of research funding for student projects (Yes)	212 (29.9%)	89 (42%)	123(58%)	**0.014**

Categorical data were presented as frequencies and percentages

Percentages are row percentages (sum to 100% across training categories)

*Statistical tests performed: Chi Square Test. Bold values indicate statistical significance (*p* < 0.05)

Regarding preferred learning methods, practical workshops (31%) was the most favored modality, while guided research projects (14%) was the least preferred, with non-trained EBM students constituting the larger proportion in each method. Finally, only 52.2% of participants reported interest in pursuing a research career, of whom 39.2% were trained in EBM.

### Search engine and sources of healthcare information retrieval

Among the total sample, PubMed/Medline was the most commonly searched database (79.3%), followed by Google (73.8%) and Google Scholar (62.1%). PubMed/Medline and Google Scholar were used significantly more by trainees (*p* value < 0.05). In contrast, usage of specialized evidence-based databases was limited overall, with Scopus (8.2%), Embase (6.1%), Cochrane Library (5.9%), and Ovid (1.0%) being the least accessed. Notably, Scopus usage was slightly higher by students trained in EBM compared to non-trainees (11.8% vs. 6.0%, *p* value = 0.007).

Regarding main sources of health information, medical books were most frequently used (83.6%), followed by scientific journals (42.6%), lecture notes (42.3%), and clinical guidelines (37.5%). Use of scientific journals (*p* = 0.006) and professional guidelines (*p* < 0.001) differed significantly based on EBM training status, with higher utilisation observed among trained students. Full results are shown in Table 3.

**Table 3:** Search engines and main sources of health information among Palestinian medical students (*N* = 709)

Variables	Overall, *N* = 709	EBM Training		*p* value*
		Yes (262)	No (447)	
Search engines used: (You can choose multiple answers)				
PubMed/Medline	562 (79.3%)	219 (83.6%)	343 (76.7%)	**0.030**
Google	523 (73.8%)	180 (68.7%)	343 (76.7%)	**0.019**
Google Scholar	440 (62.1%)	182 (69.5%)	258 (57.7%)	**0.002**
Medscape	310 (43.7%)	125 (47.7%)	185 (41.4%)	0.101
Wikipedia	219 (30.9%)	84 (32.1%)	135 (30.2%)	0.605
Web of Science	84(11.8%)	36 (13.7%)	48 (10.7%)	0.232
Scopus	58 (8.2%)	31 (11.8%)	27 (6.0%)	**0.007**
Amboss	48 (6.8%)	15 (5.7%)	33 (7.4%)	0.397
Embase	43 ( 6.1%)	18 (6.9%)	25 (5.6%)	0.492
Cochrane library	42 ( 5.9%)	19 (7.3%)	23 (5.1%)	0.251
Artificial intelligence tools	39 (5.5%)	13 (5.0%)	26 (5.8%)	0.630
Ovid	7 (1%)	3 (1.1%)	4 (0.9%)	0.745
Other	22(3.1%)	7 (2.7%)	15 (3.4%)	0.612
Main sources of health information: (You can choose multiple answers)				
Medical books	593 (83.6%)	218 (83.2%)	375 (83.9%)	0.811
Scientific journals	302 (42.6%)	129 (49.2%)	173 (38.7%)	**0.006**
Electronic media	231 (32.6%)	86 (32.8%)	145 (32.4%)	0.916
Professional guidelines	266 (37.5%)	122 (46.6%)	144 (32.2%)	**< 0.001**
Leaflets	23 (3.2%)	11 (4.2%)	12 (2.7%)	0.272
Lecture notes	300 (42.3%)	99 (37.8%)	201 (45.0%)	0.062
Opinion of health professionals	194 (27.4%)	68 (26.0%)	126 (28.2%)	0.520
Other	14 (2%)	3 (1.1%)	11 (2.5%)	0.224

Data were presented as frequencies and percentages

Percentages are calculated within each training group (column percentages)

*Statistical tests performed: Chi-Square Test. Bold values indicate statistical significance (*p* < 0.05)

### Self-reported skills in EBM

Across assessed competencies, most students rated their EBM skills as average, with fewer reporting advanced proficiency. Skills related to locating professional literature and searching online databases were predominantly rated as average, whereas critical appraisal emerged as the principal skills gap. Specifically, the two critical appraisal items showed the highest proportions of poor self-ratings (approximately 13%). Students with prior EBM training reported higher mean scores across all skill domains compared with those without prior training (*p* < 0.001 for all comparisons). Detailed distributions and group comparisons are presented in Table 4.

**Table 4:** Self-reported skills in EBM (*N* = 709)

How would you rate your skills in the following areas?	Poor	Limited experience	Average	Above Average	Advanced	EBM Training		*p * value*
						Yes	No	
Locating professional literature.	72 (10.2%)	173 (24.4%)	326 (46.0%)	110 (15.5%)	28 (3.9%)	3.18 ± 1.15	2.74 ± 1.11	**< 0.001**
Searching online databases.	41 (5.8%)	138(19.5%)	288(40.6%)	195(27.5%)	47(6.6%)	3.52 ± 1.18	3.18 ± 1.24	**< 0.001**
Critical appraisal of a scientific publication reporting findings from clinical research.	110 (15.5%)	223(31.5%)	278(39.2%)	80(11.3%)	18(2.5%)	2.89 ± 1.12	2.47 ± 1.11	**< 0.001**
Identifying knowledge gaps in practice (fields where not enough scientific literature is available to answer specific clinical question).	97(13.7%)	237(33.4%)	261(36.8%)	97(13.7%)	17(2.4%)	2.95 ± 1.18	2.54 ± 1.13	**< 0.001**
Critical appraisal of available scientific literature.	93(13.1%)	220(31.0%)	293(41.3%)	87(12.3%)	16(2.3%)	2.93 ± 1.09	2.56 ± 1.12	**< 0.001**
Identifying patient-relevant clinical questions.	56(7.9%)	181(25.5%)	287(40.5%)	152(21.4%)	33(4.7%)	3.29 ± 1.18	2.93 ± 1.21	**< 0.001**

Data were presented as frequencies and percentages (categorical), and Mean ± SD for skill scores

*Statistical tests performed: Mann-Whitney U Test. Bold values indicate statistical significance (*p* < 0.05)

### Attitudes of medical students towards using EBM in health care practice

Attitudes towards EBM were generally positive. Most students agreed or strongly agreed that evidence-based medicine is important for the practical work of physicians (73.7%) and that it improves patient care (71.8%). Additionally, 73.2% expressed a desire to further improve their EBM skills during medical training, while only 16.3% thought that evidence-based practice imposes a burden on healthcare professionals.

Mean attitude scores were generally higher in EBM-trained groups, though differences were not statistically significant. A notable exception was the perception that EBM facilitates patient-specific care, which was significantly higher among trained students (mean: 3.94 vs. 3.75, *p* value = 0.002). Table 5 summarises all attitude items.

**Table 5:** Attitudes of medical students towards using EBM in health care practice (N = 709)

Questions	Strongly disagree	Disagree	Neutral	Agree	Strongly agree	EBM Training		*p * Value*
						Yes	No	
Evidence based medicine (EBM) is important for the practical work of physicians	36 (5.1%)	26(3.7%)	124(17.5%)	266(37.5%)	257(36.2%)	4.01 ± 1.135	3.94 ± 1.028	0.092
During my studies, I would like to improve my skills in applying EBM during my practical work as a medical professional	36(5.1%)	40(5.6%)	114(16.1%)	252(35.5%)	267(37.7%)	3.97 ± 1.121	3.94 ± 1.096	0.576
EBM is important for patients to receive the optimal treatment	34(4.8%)	38(5.4%)	128(18.1%)	281(39.6%)	228(32.2%)	3.93 ± 1.141	3.87 ± 1.021	0.141
EBM facilitates decisions about individual patient’s care	25(3.5%)	39(5.5%)	168(23.7%)	284(40.1%)	193(27.2%)	3.94 ± 1.067	3.75 ± 0.966	**0.002**
EBM considers the personal expertise of physicians	40(5.6%)	69(9.7%)	271(38.2%)	231(32.6%)	98(13.8%)	3.40 ± 1.143	3.38 ± 0.949	0.449
EBM considers views and preferences of patients regarding their own therapy	36(5.1%)	90(12.7%)	278(39.2%)	218(30.7%)	87(12.3%)	3.32 ± 1.102	3.33 ± 0.957	0.839
It is important to incorporate research results into healthcare practice	32(4.5%)	45(6.3%)	134(18.9%)	271(38.2%)	227(32.0%)	3.92 ± 1.116	3.84 ± 1.049	0.141
All types of studies are of equal value	154 (21.7%)	243 (34.3%)	195 (27.5%)	87 (12.3%)	30 (4.2%)	2.48 ± 1.173	2.40 ± 1.033	0.568
EBM means an unrealistic burden to health care professionals in the daily routine patient care	95 (13.4%)	226 (31.9%)	272 (38.4%)	88 (12.4%)	28 (3.9%)	3.45 ± 1.1	3.34 ± 0.931	0.082
Textbooks are the most optimal source of information, when a question regarding the care of patients should be answered	56 (7.9%)	188 (26.5%)	271 (38.2%)	138 (19.5%)	56 (7.9%)	2.84 ± 1.055	2.98 ± 1.034	0.064
As a future healthcare practitioner, I find life-long learning as vital	35 (4.9%)	47 (6.6%)	162 (22.8%)	204 (28.8%)	261 (36.8%)	3.95 ± 1.147	3.81 ± 1.126	0.055

Data were presented as frequencies and percentages (categorical), and Mean ± SD for attitude scores

*Statistical tests performed: Mann-Whitney U Test, Bold values indicate statistical significance (*p* < 0.05)

### Knowledge of terms related to EBM

Overall, terminology knowledge showed a clear gradient with foundational concepts were better understood (e.g., sample size: 48.0% reported “understand and can explain”; prevalence and incidence: 46.0% each), whereas advanced concepts central to critical appraisal and evidence synthesis were less well understood (e.g., test power: 20.7%; heterogeneity: 19.7%; number needed to treat: 18.5%), despite many students indicating interest in further learning. Prior EBM training was consistently associated with higher mean knowledge scores across most terms (typically *p* < 0.001), including heterogeneity (3.10 ± 1.27 vs. 2.82 ± 1.20), number needed to treat (3.06 ± 1.28 vs. 2.89 ± 1.15), and meta-analysis (3.13 ± 1.36 vs. 2.84 ± 1.17). Detailed distributions and training-stratified comparisons for the knowledge items are presented in Table 6.

**Table 6:** Understanding of EBM-related terms across study design, statistics, and epidemiology domains, stratified by prior EBM training (*N* = 709)

Terms	Understand and Could explain	Some understanding	Would like	Not useful	No idea	EBM Training		*p * value*
						Yes	No	
Terms related to study design:								
Case report	355(50.1%)	257 (36.2%)	68(9.6%)	16(2.3%)	13(1.8%)	3.80 ± 1.43	3.52 ± 1.44	**0.001**
Cohort study	223(31.5%)	306(43.2%)	112(15.8%)	25(3.5%)	43(6%)	3.36 ± 1.43	2.97 ± 1.36	**< 0.001**
Randomized Controlled clinical trial	242(34.1%)	284(40.1%)	129(18.2%)	20(2.8%)	34(4.8%)	3.52 ± 1.42	3.03 ± 1.35	**< 0.001**
Meta-analysis	150(21.1%)	279(39.4%)	195(27.5%)	37(5.2%)	48(6.8%)	3.13 ± 1.36	2.84 ± 1.17	**< 0.001**
Systematic review	213(30.0%)	302 (42.6%)	133(18.8%)	24(3.4%)	37(5.2%)	3.33 ± 1.46	2.97 ± 1.29	**< 0.001**
Cross-sectional study	287(40.5%)	270(38.1%)	100(14.1%)	23(3.2%)	29(4.1%)	3.58 ± 1.45	3.26 ± 1.41	**< 0.001**
Case–control study	265(37.4%)	269(37.9%)	129(18.2%)	21(3.0%)	25(3.5%)	3.51 ± 1.40	3.22 ± 1.38	**0.003**
Terms related to statistics:								
Confidence interval	174(24.5%)	272(38.4%)	196(27.6%)	29(4.1%)	38(5.4%)	3.23 ± 1.34	2.93 ± 1.22	**< 0.001**
Sample size	340(48.0%)	228(32.2%)	99 (14.0%)	23 (3.2%)	19 (2.6%)	3.82 ± 1.37	3.49 ± 1.43	**0.015**
Mode	275(38.8%)	232 (32.7%)	141 (19.9%)	26 (3.7%)	35(4.9%)	3.56 ± 1.40	3.28 ± 1.39	**0.003**
Median	305(43.1%)	230(32.4%)	120(16.9%)	25(3.5%)	29(4.1%)	3.59 ± 1.42	3.43 ± 1.41	0.059
Interquartile range (IQR)	172(24.3%)	253(35.7%)	191(26.9%)	38(5.4%)	55(7.8%)	3.18 ± 1.34	2.94 ± 1.27	**0.005**
Standard deviation (SD)	264(37.2%)	262(37.0%)	127(17.9%)	20(2.8%)	36(5.1%)	3.48 ± 1.42	3.20 ± 1.40	**< 0.001**
Precision and accuracy	231(32.6%)	261(36.8%)	148(20.9%)	30(4.2%)	39(5.5%)	3.52 ± 1.40	3.04 ± 1.33	**< 0.001**
Representative sample	233(32.9%)	239(33.7%)	165(23.3%)	31(4.4%)	41(5.8%)	3.46 ± 1.37	3.12 ± 1.35	**< 0.001**
Test power	147(20.7%)	254(35.8%)	223(31.5%)	37(5.2%)	48(6.8%)	3.17 ± 1.29	2.86 ± 1.18	**< 0.001**
* p* value	241(34.0%)	237(33.4%)	158(22.3%)	38(5.4%)	35(4.9%)	3.47 ± 1.34	3.20 ± 1.37	**0.002**
Type I and type II errors	200(28.2%)	239(33.7%)	191(26.9%)	39(5.5%)	40(5.6%)	3.29 ± 1.34	3.10 ± 1.29	**< 0.001**
Terms related to Epidemiology:								
Relative risk	212(29.9%)	268(37.8%)	160(22.6%)	32(4.5%)	37(5.2%)	3.40 ± 1.38	3.02 ± 1.30	**< 0.001**
Absolute risk	208(29.3%)	269(37.9%)	166(23.4%)	33(4.7%)	33(4.7%)	3.37 ± 1.38	3.04 ± 1.28	**< 0.001**
Odds ratio	198(27.9%)	268(37.8%)	169(23.8%)	33(4.7%)	41(5.8%)	3.36 ± 1.37	2.96 ± 1.28	**< 0.001**
NNT (number needed to treat)	131(18.5%)	237(33.4%)	237(33.4%)	50(7.1%)	54(7.6%)	3.06 ± 1.28	2.89 ± 1.15	**< 0.001**
Sensitivity of a diagnostic test	271(38.3%)	234(33.0%)	135(19.0%)	35(4.9%)	34(4.8%)	3.54 ± 1.40	3.30 ± 1.39	**< 0.001**
Specificity of a diagnostic test	274(38.6%)	243(34.3%)	132(18.6%)	26(3.7%)	34(4.8%)	3.55 ± 1.43	3.27 ± 1.39	**< 0.001**
Heterogeneity	140(19.7%)	272(38.4%)	206(29.1%)	40(5.6%)	51(7.2%)	3.10 ± 1.27	2.82 ± 1.20	**< 0.001**
Publication bias	200(28.2%)	266(37.5%)	160(22.6%)	37(5.2%)	46(6.5%)	3.28 ± 1.37	3.01 ± 1.32	**< 0.001**
Lost to follow-up	190(26.8%)	253(35.7%)	163(23.0%)	45(6.3%)	58(8.2%)	3.23 ± 1.37	2.99 ± 1.33	**< 0.001**
Randomization	285(40.2%)	249(35.1%)	116(16.4%)	23(3.2%)	36(5.1%)	3.57 ± 1.43	3.28 ± 1.42	**< 0.001**
Intention-to-treat analysis	123(17.3%)	243(34.3%)	241(34.0%)	41(5.8%)	61(8.6%)	3.13 ± 1.30	2.75 ± 1.11	**< 0.001**
Prevalence	326(46.0%)	221(31.2%)	102(14.4%)	27(3.7%)	33(4.7%)	3.81 ± 1.41	3.40 ± 1.44	**< 0.001**
Incidence	326(46.0%)	228(32.2%)	92(13.0%)	27(3.7%)	36(5.1%)	3.78 ± 1.42	3.39 ± 1.46	**< 0.001**
Positive predictive value	258(36.3%)	248(35.0%)	134(18.9%)	33(4.7%)	36(5.1%)	3.54 ± 1.42	3.20 ± 1.37	**< 0.001**
Hierarchy of evidence	151(21.3%)	220(31.1%)	229(32.3%)	47(6.6%)	62(8.7%)	3.20 ± 1.36	2.89 ± 1.18	**< 0.001**
Clinical effectiveness	182(25.7%)	264(37.2%)	189(26.7%)	30(4.2%)	44(6.2%)	3.29 ± 1.38	2.92 ± 1.23	**< 0.001**
Practical guideline	202(28.6%)	261(36.8%)	162(22.8%)	37(5.2%)	47(6.6%)	3.40 ± 1.41	2.96 ± 1.28	**< 0.001**
Evidence-based medicine	241(34.1%)	271(38.2%)	123(17.3%)	37(5.2%)	37(5.2%)	3.69 ± 1.43	2.99 ± 1.31	**< 0.001**

Data were presented as frequencies and percentages (categorical), and Mean ± SD for knowledge scores

*Statistical tests performed: Mann-Whitney U Test, Bold values indicate statistical significance (*p* < 0.05)

## Discussion

Evidence-based medicine is increasingly recognized as an important component of modern medical practice, employing physicians expertise, best available evidence into patient centered medical care. Its inclusion into healthcare curriculum has been sought worldwide, yet its implementation remains weak in low- and middle- income countries. This study evaluated EBM-related knowledge, self-reported skills, attitudes, and educational exposure among Palestinian medical students across multiple universities and training stages.

Our study found that a significantly higher proportion of participants who attended biostatistics or research methodology courses had prior EBM training. In addition, students held positive perceptions of EBM, with the majority agreeing that it improves patient care and informs clinical decision-making, with the latter having a significant higher mean score among EBM-trained students. Interestingly, although participants expressed moderate self-confidence when rating their EBM skills, those who had prior training scored higher self-rating in each skill domain compared to non-trained peers (*p* value < 0.001). This finding suggests an association between prior exposure to EBM training and higher self-reported confidence in performing EBM-related tasks. Our findings are comparable to those of previous cross-sectional studies in various settings. For instance, a survey among German and British surgeons found that most participants considered EBM as an important component for patient-centered care and decision making process [20]. Similarly, a cross sectional study among physicians in Gaza reported that over 70% welcomed the integration of EBM into practice, although younger doctors (below 30) were more likely to consider it as an extra burden to their workload compared to older doctors (above 30) (*p* value = 0.0176) [14]. In contrast, a study conducted in Saudi Arabia showed unfavourable stance towards EBM among medical undergraduates [21].

In regard to self-perceived skills, Csertő et al. [5] found that medical students with previous exposure to EBM courses rated their searching and evaluating abilities significantly higher compared to students with no prior exposure. On the other hand, a Chinese hospital-based study found an overall low self-rated skills among physicians, despite their recognition of EBM importance. The authors also found that medical background, attitude and hospital’s research requirements accounted for 22.9% of the variance in EBM skills based on a multivariate regression analysis [22]. Another study conducted among sudanese medical undergraduates highlighted similar results. Participants reported limited experience in most skill domains based on the same questionnaire applied in our study. However, the mean scores were significantly higher among EBM-trained students compared to non-trained, findings consistent with our results [19]. These varying findings across studies could be attributed to the variation of participants (physicians and students), educational systems, cultural settings and assessment methods employed.

Basic epidemiological and statistical concepts were better understood by a larger proportion of students in our study, whereas advanced concepts such as heterogeneity, and type I\II errors were less familiar to participants. This is consistent with a recent sudanese study that reported high understanding among medical students for basic concepts such as sample size, prevalence and incidence, while intention-to-treat analysis and type I\II errors were less understood with 19% and 19.3% of students, respectively, being able to explain these terms [12]. Additionally, Meta-analysis design was only understood by 19.9% of participants which is close to our results, in which 20.1% reported good understanding of its concept. Similar trends were observed by Csertő et al. with students who trained in EBM having significantly better general knowledge about such terms than those who had no training [5]. Our results are further supported by a Syrian study, which delivered a two-day intensive EBM course to medical students at Damascus University. Authors found that knowledge about PICO formulation, statistics handling and sample size calculation were low before training but increased significantly post-training with improvements ranging from 35.3% to 56.9% (*p* value < 0.001). Interestingly though, when knowledge was compared to objective assessment, students tended to overestimate their practical understanding (28% vs. 17.86%) [23].

The inverse association between clinical training and prior EBM training likely reflects implementation constraints in busy clinical environments rather than a lack of perceived value. In a scoping review of EBM curricula for physicians in training, time was identified as the primary barrier to EBM curriculum implementation [24]. Clinical clerkships are often considered an ideal setting for clinically integrated EBM, yet EBM teaching is frequently not optimally enacted during rotations because learners struggle to translate classroom-acquired EBM skills into real-time clinical decision-making without deliberate workplace-based reinforcement [25]. Evidence from hospital-based training similarly suggests that higher clinical workload/patient load reduces available time, which is described as a major barrier to EBM engagement [26]. In addition, program-level data indicate that limited faculty development and limited faculty time/skill to teach EBM constrain effective EBM instruction in clinical training settings [27].These mechanisms provide a plausible explanation for why students engaged in hospital-based clinical training may report lower exposure to formal EBM training, particularly when EBM teaching is concentrated in earlier structured coursework and not systematically embedded during rotations.

To provide additional insight into the educational context influencing EBM learning in Palestine, resource utilisation was assessed. Our study showed that search engines such as PubMed, Google and Google Scholar were widely utilised, with higher proportion of users for PubMed and Google Scholar being in the EBM-trained group. On the other hand engagement with specialized medical databases remained overall limited. For example, only 8.2% reported searching Scopus, though its usage was slightly higher among EBM-trainees compared to non-trainees (11.8% vs. 6.0%, *p* value = 0.007). This could be explained by the lack of access to subscription-based databases through affiliated institutions as 80.8% reported having no access, highlighting resource inequities in Palestine. Consequently, medical textbooks were the primary source of health information for 83.6% of participants, whereas scientific journals and professional guidelines were less frequently used. Interestingly, among students who used journals and guidelines, a higher proportion were trained in EBM. Similarly, Hungarian EBM-trained students demonstrated greater use of online journals and professional guidelines (*p* value < 0.001), although printed books were the most relied-on overall. As for Cochrane database usage was more frequent among trained students but remained low overall at 5.02% [5]. Jaber Amin et al., also found that sudanese medical students relied primarily on textbooks and that specialized databases such as Cochrane were significantly more used by trained students, though overall access was similarly low [12]. These differences could be attributed to multiple factors, including the proportion of EBM-trained students in each study, limited institutional funding and support, variation in curricular structures and resources accessibility. This is supported by a systematic review on barries to accessing scholarly resources in LMICs, which found that time constraints, and limited institutional funding and support restrict accessing high-quality resources and hinder proper implementation of evidence-based medicine in such countries [28].

Despite broadly favourable attitudes toward EBM, our results indicate a clear attitude-competency gap; students consistently endorsed EBM’s relevance to patient care, yet self-assessed skills clustered largely in the average range, with critical appraisal emerging as the dominant skill deficit, reflected by the highest proportion of poor ratings on appraisal-related tasks. In parallel, terminology knowledge was stronger for basic concepts than advanced concepts, indicating limitations in the interpretive knowledge needed to appraise and apply evidence in clinical decisions. This pattern mirrors prior undergraduate data showing that learners may value EBM but still report limited experience with critical appraisal, and that prior EBM coursework is associated with higher self-reported searching/appraisal skills [19]. Accordingly, the most pressing targets for curricular enhancement are critical appraisal and interpretation (Appraise) and efficient retrieval of high-quality evidence (Acquire), particularly given students’ predominant reliance on textbooks and the comparatively limited use of specialized evidence resources.

Curriculum-level implications are best articulated by mapping our results to the five EBM steps (Ask, Acquire, Appraise, Apply, Assess), a widely used organizing framework for EBM competency and evaluation [29]. In our study, the most pronounced deficits clustered within Acquire (limited uptake of higher-quality evidence sources beyond textbooks) and Appraise (appraisal tasks as the most consistent skill deficit). These patterns support a staged, vertically integrated approach; preclinical curricula should prioritize structured question formulation (Ask; e.g., PICO) and efficient evidence retrieval (Acquire; database navigation, filters/MeSH, and guideline repositories), while clinical clerkships should concentrate on repeated, supervised practice in critical appraisal and interpretation (Appraise) and structured application to authentic patient scenarios (Apply). The Assess step can be operationalized through low-burden clerkship routines such as brief reflective notes, outcome-focused follow-up questions, and simple audit-and-feedback sessions to support ongoing learning and encourage continued EBM use.

In resource-constrained settings, EBM instruction should focus on scalable, low-cost approaches that can be delivered within existing teaching structures. Evidence acquisition can be strengthened through open-access resources paired with structured search exercises, reducing reliance on informal, non-curated information sources under clinical time pressure. Competency development can then be reinforced through brief, context-driven, case-based tasks embedded in tutorials, ward rounds, or structured post-rotation teaching sessions. A pragmatic model is a “case-triggered” activity in which students formulate a focused clinical question, conduct a targeted search, critically appraise one relevant study using a standardized checklist, and translate findings to the index patient. Finally, a short, structured EBM module combining concise didactic content with hands-on workshops and supervised search practice may offer a sustainable pathway to strengthen EBM skills while remaining feasible within low-resource medical education systems.

## Strengths and Limitations

Our study has several strengths, including a large multi-institutional sample and the use of validated EBM instruments. This strengthens the robustness of our results and provides a broad overview of EBM-related knowledge, skills, and attitudes among Palestinian medical students. Although convenience sampling may limit representativeness, this diversity of participating institutions allows our findings to serve as a reference for similar educational settings within Palestine and other neighboring low-income countries.

As for study limitations. First, the cross-sectional design hinders assessing causation between EBM training status and knowledge and skills. Second, the subjective nature of the questionnaire may not fully reflect actual competency practical skills of students. Additionally, detailed information regarding the extent, format, and duration of prior EBM training was not collected which warrants cautious interpretation of our results.

Importantly, recruitment relied on online convenience sampling via academic social media groups, which may introduce selection and self-selection bias. Students who are more motivated, research-oriented, or already exposed to EBM may have been more likely to participate, potentially inflating estimates of positive attitudes, self-reported skills, and training exposure; digital distribution may also underrepresent students with lower engagement in online platforms or inconsistent connectivity. These factors may limit generalizability to all Palestinian undergraduate medical students.

Future research should assess the EBM concepts and skills using objective measures and retrieve details regarding EBM training to better evaluate proficiency and the effectiveness of educational interventions among Palestinian medical students.

## Conclusion 

Palestinian medical students demonstrate generally positive attitudes towards evidence-based medicine, though self-reported confidence in their skills was moderate. Furthermore, meaningful gaps persist in understanding advanced EBM-related terminology, and effective information retrieval. This reflects structural educational challenges, including limited access to subscription-based resources, inconsistent academic mentorship, and insufficient opportunities for hands-on formal EBM training. Strengthening EBM education through curricular integration, faculty support initiatives, and improved access to scientific resources may assist in addressing such gaps.

## Data Availability

The datasets used and/or analysed during the current study are available from the corresponding author upon reasonable request.
